# Genetic influences on attention deficit hyperactivity disorder symptoms from age 2 to 3: A quantitative and molecular genetic investigation

**DOI:** 10.1186/1471-244X-10-102

**Published:** 2010-12-01

**Authors:** Nicholas E Ilott, Kimberly J Saudino, Philip Asherson

**Affiliations:** 1 SGDP Research Centre, Institute of Psychiatry, Kings College, London, UK; 2 Psychology Department, Boston University, 64 Cummington St., Boston, MA, USA

## Abstract

**Background:**

A twin study design was used to assess the degree to which additive genetic variance influences ADHD symptom scores across two ages during infancy. A further objective in the study was to observe whether genetic association with a number of candidate markers reflects results from the quantitative genetic analysis.

**Method:**

We have studied 312 twin pairs at two time-points, age 2 and age 3. A composite measure of ADHD symptoms from two parent-rating scales: The Child Behavior Checklist/1.5 - 5 years (CBCL) hyperactivity scale and the Revised Rutter Parent Scale for Preschool Children (RRPSPC) was used for both quantitative and molecular genetic analyses.

**Results:**

At ages 2 and 3 ADHD symptoms are highly heritable (*h*^*2 *^*= *0.79 and 0.78, respectively) with a high level of genetic stability across these ages. However, we also observe a significant level of genetic change from age 2 to age 3. There are modest influences of non-shared environment at each age independently (*e*^*2 *^= 0.22 and 0.21, respectively), with these influences being largely age-specific. In addition, we find modest association signals in *DAT1 *and *NET1 *at both ages, along with suggestive specific effects of *5-HTT *and *DRD4 *at age 3.

**Conclusions:**

ADHD symptoms are heritable at ages 2 and 3. Additive genetic variance is largely shared across these ages, although there are significant new effects emerging at age 3. Results from our genetic association analysis reflect these levels of stability and change and, more generally, suggest a requirement for consideration of age-specific genotypic effects in future molecular studies.

## Background

Attention Deficit Hyperactivity Disorder (ADHD) is a common neurodevelopmental disorder characterised by pervasive, age inappropriate behaviours of inattention, hyperactivity and impulsivity. The current definition of ADHD defines the age of onset of impairing symptoms as occurring before the age of 7 years, although formal diagnoses are not usually made before this age. However, early characteristics are good predictors of later appearing behavioural problems [1] and therefore, employing research strategies to identify developmental aetiological factors in young children remains important. It is well established that ADHD in children is highly heritable with estimates averaging at ~76% [2], with the same being true of ADHD symptoms in pre-school children [3]. However, genetic variation underlying these observed heritabilities is still not well understood.

Candidate gene studies in children have focused predominantly on genes of monoaminergic neurotransmitter systems, particularly dopamine. The main genes of interest in this research have been the dopamine transporter gene (*DAT1*) and dopamine receptor genes (*DRDs*). These choices have been informed by a dopamine hypothesis of ADHD, which stems from the action of stimulant medications such as methylphenidate and dexamphetamine which increase levels of available synaptic dopamine. These studies have proven relatively fruitful with robust associations between *DRD4 *and *DRD5 *with ADHD being identified in meta-analysis [4]. More recently, whole genome association analyses in both children and adults have provided some information on potential new candidates for follow up [5-7]. Of particular interest is the convergent finding of association with variants within *CDH13*, a gene that lies within the ADHD linkage region on chromosome 16p [5,8]. This has provided new insights into the underlying genetics of ADHD and has allowed for new hypotheses to be formed for future research. However, there have been fewer molecular studies in preschool children, although there is some evidence to suggest that candidate genes from various neurotransmitter systems such as *DAT1*, synaptosome-associated Protein 25 (*SNAP25*) and the noradranaline transporter (*NET1*) may have some involvement [9].

It is apparent that these genes are not necessarily acting on the ADHD phenotype consistently throughout development, with a number of studies suggesting that although there is a general genetic stability across time from ages 2 through to 4 years [10]; 2, 3, 4 and 7 years [11]; 3 through 12 years [12] and 8 through to 14 years [13], there is also age-specific genetic variance. The implications of this are that association studies using heterogeneous samples are potentially losing information on age-specific effects of genotype on ADHD. Further, with the need for replication across studies it becomes very difficult to identify the causes of non-replication due to differences in sample demographics.

We have recently reported high heritability and genetic association between specific risk alleles and ADHD symptom scores in a population sample of 2-year old twins, with modest evidence of association being found for *DAT1 *and *NET1 *[14]. In the present analysis we have used the same sample to assess the degree to which genetic effects on ADHD symptoms are stable from ages 2 to 3 using quantitative genetic techniques. In addition to this analysis, we have studied previously reported ADHD risk alleles to identify any age-specific genetic associations. Candidate gene variants were chosen based on previous positive association with ADHD in either clinical or quantitative trait locus (QTL) analyses. Given the nature of the analyses we hypothesised that there would be substantial genetic overlap in ADHD symptom scores across ages, which would translate into a number of genetic variants at age 2 also being associated at age 3.

## Method

### Sample

The Boston University Twin Project sample was recruited from birth records supplied by the Massachusetts Registry of Vital Records. Ethical approval was obtained for the study through the joint South London and Maudsley and the Institute of Psychiatry NHS Research Ethics Committee ref. 2002/238. Twins were selected preferentially for higher birth weight and gestational age. No twins with birth weights below 1750 grams or with gestational ages less than 34 weeks were included in the study. Twins were also excluded if one or both twins had a health problem that might affect motor activity (e.g., cerebral palsy, club foot) or had chromosomal abnormalities. The present analyses include 312 same-sex pairs of twins (144 MZ, 168 DZ; 164 male pairs, 148 female pairs). Although the sample was predominately Caucasian (85.4%), ethnicity was generally representative of the Massachusetts population (3.2% Black, 2% Asian, 7.3% Mixed, 2.2% Other). Socioeconomic status according to the Hollingshead Four Factor Index (1975) ranged from low to upper middle class (range = 20.5-66; *M *= 50.9, *SD *= 14.1).

Zygosity was determined via DNA analysis using DNA obtained from cheek swab samples. In the cases where DNA was not available (*n *= 3), zygosity was determined using parents' responses on physical similarity questionnaires which have been shown to be more than 95% accurate when compared to DNA markers [15]. In our present sample we were able to assign zygosity with certainty to 99% of the twin pairs using the parent questionnaire, moreover agreement between questionnaire and DNA zygosity analyses was very high (kappa = .94).

### Parent Reports of ADHD Behaviour

Written informed consent was obtained from parents and they were invited to assess their children's behaviour at two time points; 1) within two weeks of their second birthday and 2) within two weeks of their third birthday. The mean age at time point 1 was 2.07 years (*SD *= 0.05) and at time point 2 it was 3.05 (*SD *= 0.05).

Parent ratings of hyperactivity were obtained from either parent using the hyperactivity subscales of the Child Behavior Checklist/1.5 - 5 years (CBCL) [16] and the Revised Rutter Parent Scale for Preschool Children (RRPSPC) [17] which assess behaviors relating to overactivity, inattention, and impulsivity. Of the total sample 94% mothers and 6% fathers completed the questionnaires, with the same parent completing the questionnaire at both ages. In the present study reliabilities for the CBCL and the RRPSPC, as estimated by Cronbach's alpha were .78 and .75, respectively. The two ADHD measures correlated significantly at both time points (age 2, *r = 0.67*, p < 0.01 and age 3, *r = 0.65*, p < 0.01; data based on 312 individuals). These measures also display high genetic correlations at both ages (age 2 *rG = *0.71, age 3 *rG = *0.76, analyses are available on request from first author). Scores from these measures were subsequently averaged to form an ADHD composite measure, which was square root transformed for a more normal distribution.

### Model Fitting Analysis

Because twin co-variances can be inflated by variance due to sex, all scores were residualised for sex effects. Residualised scores were used for all model fitting procedures.

A Cholesky decompositon model was used to estimate the relative contributions of additive genetics (A), shared environment (C) and non-shared environment (E) to the phenotypic variance of ADHD at each age, as well as genetic and environmental contributions to the co-variation between ages. Models were fit to raw data using a maximum likelihood pedigree approach implemented in Mx structural equation modelling software [18]. The overall fit of a model was assessed by calculating twice the difference between the negative log-likelihood (-2LL) of the model and that of a saturated model (i.e., a model in which the variance/covariance structure is not estimated and all variances and covariances for MZ and DZ twins are estimated).

### Genotyping

Polymorphisms were chosen based on previous association with ADHD in either clinical or QTL studies (Table 1). DNA was extracted from buccal swabs as described by Freeman *et al*. 2003[19]. Both parents and offspring were genotyped. VNTR polymorphisms (*DRD4 *exon 3, *DAT1 *3'UTR, *DAT1 *intron 8, the *5-HTT*LPR and *MAOA *promoter) were genotyped in-house. Protocols for genotyping the VNTRs are available on request from the authors. Single nucleotide polymorphisms (SNPs) were genotyped by Prevention Genetics http://www.preventiongenetics.com/resgeno/researchgeno.htm.

**Table 1:** Genetic markers chosen for genotyping and position in the genome (chromosome and respective chromosomal position in bp) based on UCSC May 2004 Human assembly.

*Gene*	*Marker*	*Chromosome*	*Position (bp)*
*DRD4*	*rs1800955*[26]	11	626,534
	*rs747302*[26]	11	626,439
	Exon 3 VNTR[4,27,28]	11	629,989-630,194

*DAT1*	3'UTR VNTR[29]	5	1,446,697-1,447,100
	*rs40184*[30]	5	1,448,327
	*rs3776513*[30]	5	1,459,854
	Intron 8 VNTR[30,31]	5	1,464,856-1,465,037
	*rs2042449*[30]	5	1,469,396
	*rs2652511*[30]	5	1,499,139
	rs11564750[30]	5	1,501,012
	rs2550946[30]	5	1,503,763

*SNAP25*	rs6039806[32]	20	10,206,904
	rs362987[32]	20	10,225,702
	rs3746544[33-35]	20	10,235,334
	rs1051312[33,34]	20	10,235,338

*5-HTT*	rs11080121[36]	17	25,553,218
	rs140701[37]	17	25,562,908
	rs2020936[36]	17	25,574,940
	rs2066713[36]	17	25,576,041
	*rs1050565*[37]	17	25,599,952
	5-HTTLPR[37]	17	25,588,361 - 25,588,889

*MAOA*	rs6323[37,38]	X	43,347,040
	Promoter VNTR[39,40]	X	43,270,603 - 43,270,707

*NET1*	rs11568324[30,41]	16	54,283,809
	rs3785157[42,43]	16	54,287,587
	rs998424[42,43]	16	54,289,697
	rs2242447[43]	16	54,293,663

*TPH2*	*rs1843809*[30,44]	12	70,635,215
	*rs1386493*[30,44]	12	70,641,196
	*rs1386497*[30,44]	12	70,678,307

Various genotyping quality control measures were implemented to assess the impact of potential error. Mendelian discrepancies in the data were checked using PEDSTATS http://www.sph.umich.edu/csg/abecasis/QTDT/download/[20]. The average Mendelian error rate for the VNTR genotyping was 0.65% with the highest rate being for the MAOA promoter VNTR (1.45%). Where inheritance errors were detected, genotypes for that family were coded '0'.

Eight of the chosen SNPs (rs3776513, rs2042449, rs1386493, rs1386497, rs1050565, rs2652511, rs1800955 and rs747302) failed at the stage of assay design. For the remaining 17 SNPs the average Mendelian error rate was 1.05%. A breakdown by SNP revealed two SNPs, rs40184 and rs1843809 that had high Mendelian error (2.03% and 8.39%, respectively) and these two SNPs were omitted from further analysis. With these SNPs removed, the error rate was reduced to 0.47% and remaining inheritance errors were coded as missing genotypes for the family/genotype combination. A second genotyping control measure was the use of a sex specific marker. The error associated with sex anomalies was 0.35%. Along with the specific sex marker, genotyping error on X-linked markers (MAOA promoter VNTR and rs6323) gave an additional sex discrepancy error of 0.008%. A further quality control measure was through genotyping 96 random duplicates. Only 0.02% of duplicated samples were not consistent with the original genotype. Taken together, genotyping error was estimated to be 1.5% plus hidden error. Hidden error can be considered as 1/3 total genotyping error. With additional, hidden genotyping error included, the genotype error rate including both detected and undetected errors may be as high as 4.5%. All markers included in the analysis conformed to Hardy-Weinberg equilibrium (p > 0.01).

### Association Analysis

Tests of allelic association were performed using the Quantitative Transmission Disequilibrium Test (QTDT) [20] on ADHD scores residualised for sex effects. An advantage of using QTDT in association analyses using twin data is that all families remain informative regardless of twin class. QTDT tests for association in a variance components framework and using the -weg command in the program, one can model the phenotypic similarities that are due to sharing of the genome (polygenic (g), 100% for MZ twins and 50% for DZ twins), as well as phenotypic differences that are due to non-shared environmental influences (e). Three models of association were tested using a likelihood ratio test implemented in QTDT: the 'Total Association' test (AT), the 'Within-Test' of association (AW) and the test of stratification (AP). These different models provide the user with varied information regarding association statistics and tests of stratification. Overall association was tested using the AT model which assesses both the within-pair differences as well as between-pair sums (i.e. the correlation between phenotypic and genotypic differences and sums for each twin pair) and is the most powerful test in the absence of stratification effects. In contrast, the AW assesses the within component only. The within-pair design of the AW means that it is unaffected by between-family stratification effects, yet is less powerful than the AT in the absence of stratification. Based on the differences between these two models, the significance of association should consider stratification effects. To evaluate this we modelled association using the AP test which compares the significance from the between component versus the within component of association. Stratification effects are dismissed when these components are equal and p > 0.05. In this instance, results are interpreted from the AT. Conversely, results are interpreted from the AW if significant stratification effects are detected. VNTR markers were tested using the 'multi-allelic' function in QTDT. This provides a single p-value for tests of alleles with an allele frequency >0.05.

UNPHASED http://www.mrc.bsu.cam.ac.uk/personal/frank/software/unphased/ was used to test X-linked markers (polymorphisms in *MAOA*) because QTDT cannot deal with such data. Because UNPHASED has no means for handling MZ twin data, mean phenotypic scores for MZ pairs were used in these analyses.

## Results

Descriptive statistics for the measures analysed in this sample are presented in Table 2. Intraclass correlations at both ages displayed DZ correlations that were roughly half MZ correlations, inferring predominantly additive genetic effects (Table 3). When compared to a saturated model, the fit of the data to the Cholesky decomposition model was not significantly different (χ^2 ^= 13.85, df = 11, p = 0.24, Table 4). The majority of the variance for ADHD symptoms at ages 2 and 3 was explained by additive genetic influences, producing estimates for A of 0.78 (95%CI 0.65 - 0.83) and 0.79 (95%CI 0.65 - 0.84) (Table 3), respectively. There were no significant effects of C on the trait variance at either age (Table 3), with no detriment in fit when this parameter was dropped from the model (χ^2 ^= 0, df = 3). There were modest effects of E at both ages (age 2, E = 0.22, 95%CI 0.17 - 0.29 and age 3, E = 0.21, 95%CI 0.16 - 0.27).

**Table 2:** Descriptive statistics for ADHD scale raw scores.

	*CBCL ADHD scale*	*RRPSPC scale*	ADHD Composite*
*Mean (age 2)*	4.28	2.10	1.21
*SD (age 2)*	2.57	1.89	0.37
			
*Mean (age 3)*	3.99	2.12	1.16
*SD (age 3)*	2.64	1.91	0.39

N = 312; each mean and SD calculated through a random selection of one twin from each pair. *Scores residualised for sex effects and square-root transformed.

**Table 3:** Intraclass correlations (95%CI) and variance components estimates (95%CI).

	*rMZ*	*rDZ*	A	C	E
*ADHD*					
*Composite Age 2*	0.77 (0.69 - 0.83)	0.34 (0.19 - 0.47)	0.79 (0.65 - 0.84)	0.00 (0.00 - 0.11)	0.21 (0.16 - 0.27)

*ADHD*					
*Composite Age 3*	0.74 (0.65 - 0.80)	0.32 (0.17 - 0.45)	0.78 (0.65 - 0.83)	0.00 (0.00 - 0.13)	0.22 (0.17 - 0.29)

**Table 4:** Fit statistics for the overall fit of the longitudinal Cholesky decomposition model.

	Overall Fit of Model					
**Model**	**-2LL**	***df***	**Δχ**^**2**^	**Δ*df***	***AIC***	***p***

*Saturated*	484.49	1177				
*Cholesky decomposition*	498.34	1189	13.85	11	-8.15	0.24

**Figure 1:**
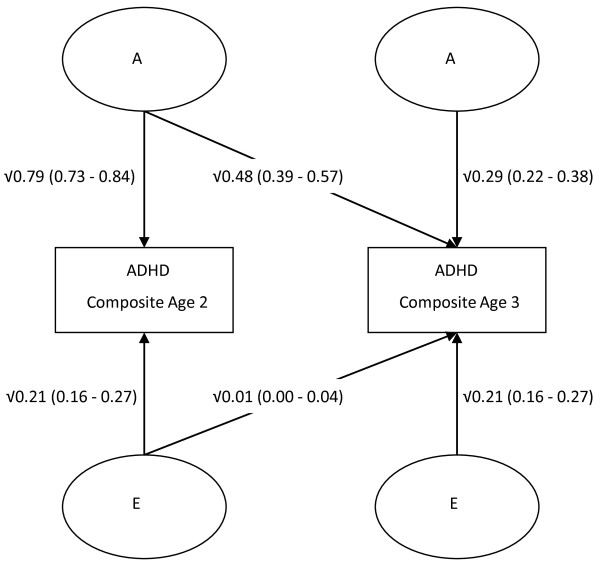
**Cholesky decomposition model showing influences of A (additive genetics) and E (non-shared environment) on the variance and covariance of ADHD symptoms across age 2 and 3**. Squared path estimates (95%CI) are provided.

From the Cholesky decomposition model (Figure 1) we can estimate the degree to which A, C and E contribute to the co-variance of ADHD symptoms across time. C has been omitted from Figure 1 because of the lack of significant C on the variance at either age. All path estimates are provided from the most parsimonious AE model.

A large proportion of the additive genetic variance at age 2 was shared with that at age 3 (Figure 1), although there remained emerging age-specific effects (Figure 1). Indeed, dropping the age 3-specific A path from the Cholesky decomposition model resulted in a significant worsening in fit (χ^2 ^= 12.263, df = 1, p < 0.01), suggesting a contribution of genetics to both phenotypic stability and change. The effect of E on the covariation between ages was small, yet significant (Figure 1). Using unsquared path estimates from the Cholesky decomposition model, we can estimate the correlation between ADHD symptoms at age 2 and 3. In this case the phenotypic correlation between ages is calculated as (√0.79 × √0.48) + (√0.21 × √0.01) = 0.67. Additive genetic influences account for 93% of this correlation (bivariate heritability = ((√0.79 × (*rG = *0.78) × √0.79)/0.67) × 100 = 93%).

### Molecular Genetic Analysis

#### Total Test of Association (AT)

At age 2, nominal association was detected between the *DAT1 *3'UTR VNTR (χ^2 ^= 7.00, df = 2, p = 0.03) and one *NET1 *SNP, rs11568324 (χ^2 ^= 4.38, df = 1, p = 0.04) with the ADHD composite (Table 5). Two additional SNPs in *NET1*, rs3785157 (χ^2 ^= 3.68, df = 1, p = 0.06) and rs998424 (χ^2 ^= 3.30, df = 1, p = 0.07) and a SNP in *5-HTT*, rs140701 (χ^2 ^= 2.96, df = 1, p = 0.09) provided weak evidence of association with this measure (Table 5).

**Table 5:** QTDT analysis.

		*ADHD Composite Age 2*						*ADHD Composite Age 3*					
***Gene***	***Marker***	***AT***			***AW***			***AT***			***AW***		
		
		***χ***^***2***^	***df***	***P***	***χ***^***2***^	***df***	***P***	***χ***^***2***^	***df***	***p***	***χ***^***2***^	***df***	***p***

*DRD4*	Exon 3 VNTR	4.74	3	0.19	3.26	3	0.35	7.82	3	***0.05***	8.69	3	***0.03***

*DAT1*	3'UTR VNTR	7.00	2	***0.03***	5.09	2	*0.08*	11.15	2	***0.004***	12.17	2	0.002*
	Int8 VNTR	3.42	2	0.18	2.84	2	0.24	2.90	2	0.24	3.21	2	0.20
	rs11564750	0.06	1	0.80	0.75	1	0.39	0.14	1	0.43	0.01	1	0.94
	rs2550946	0.43	1	0.51	0.71	1	0.40	0.16	1	0.47	0.99	1	0.32

*SNAP25*	rs6039806	0.00	1	0.96	0.00	1	0.99	0.08	1	0.97	0.41	1	0.52
	rs362987	0.04	1	0.84	0.07	1	0.79	0.04	1	0.91	0.39	1	0.39
	rs3746544	0.05	1	0.83	0.18	1	0.67	1.75	1	0.21	1.98	1	0.16
	rs1051312	0.63	1	0.43	0.04	1	0.85	0.00	1	0.58	0.65	1	0.42

*5-HTT*	rs11080121	2.16	1	0.14	2.71	1	0.10	0.12	1	0.23	4.77	1	***0.03***
	rs140701	2.96	1	*0.09*	3.03	1	*0.08*	0.00	1	0.38	3.24	1	***0.07***
	rs2020936	1.24	1	0.27	1.92	1	0.17	1.34	1	0.40	1.20	1	0.27
	rs2066713	0.04	1	0.84	0.00	11	1.00	1.27	1	0.50	0.32	1	0.57
	5-HTTLPR	0.31	1	0.57	0.03	1	0.87	0.03	1	0.87	0.91	1	0.34

*MAOA*	rs6323	NT	NT	NT	1.03	1	0.31	NT	NT	NT	0.97	1	0.83
	Promoter VNTR	NT	NT	NT	4.68	3	0.20	NT	NT	NT	0.05	3	0.81

*NET1*	rs11568324	4.38	1	***0.04***	NT	NT	NT	0.58	1	0.13	NT	NT	NT
	rs3785157	3.68	1	*0.06*	4.65	1	***0.03***	0.37	1	0.48	4.30	1	***0.04***
	rs998424	3.30	1	*0.07*	4.42	1	***0.04***	0.83	1	0.59	3.22	1	*0.07*
	rs2242447	1.23	1	0.27	1.03	1	0.31	3.56	1	0.18	2.01	1	0.16

Nominal p-values < 0.05 are in **bold**, *italicized *numbers and those approaching this significance threshold are shown in *italics*. AT = Total Test of Association, AW = Within-Test of Association. NT = Not tested. X-linked markers tested using UNPHASED. df = difference in degrees of freedom between the null and alternative models. *Significant after bonferroni correction

At age 3, nominal association was detected between the same *DAT1 *polymorphism (χ^2 ^= 11.15, df = 2, p = 0.004) as at age 2, as well as the *DRD4 *exon 3 VNTR (χ^2 ^= 7.82, df = 3, p = 0.05).

Given the non-independent nature of the phenotypes under investigation, we did not correct any of the association findings for the number of phenotypes studied. None of the associations at either age withstood Bonferroni correction for 20 comparisons (20 markers) at p < 0.05.

#### Within Test of Association

At age 2 we found no evidence for stratification effects (AP test, data not shown), although it cannot be ruled out due to low power to detect it in this sample. We therefore completed the AW test for all genetic markers, which is robust to stratification effects. Two SNPs in *NET1*, rs3785157 (χ^2 ^= 4.65, df = 1, p = 0.03) and rs998424 (χ^2 ^= 4.42, df = 1, p = 0.04) showed nominal significance in this test with the ADHD composite, although high linkage disequilibrium (LD) between these SNPs suggests non-independence. Further, the *DAT1 *3'UTR VNTR (χ^2 ^= 5.09, df = 2, p = 0.08) and rs140701 (χ^2 ^= 3.03, df = 1, p = 0.08) displayed an association trend with the same measure (Table 5).

At age 3 we found evidence for stratification in the AP test for two markers in *NET1*, rs3785157 and rs998424 (χ^2 ^= 5.42, df = 1, p = 0.02 and χ^2 ^= 4.46, df = 1, p = 0.03, respectively). Nominal associations were found with rs3785157 in *NET1 *(χ^2 ^= 4.30, df = 1, p = 0.04), rs11080121 in *5-HTT *(χ^2 ^= 4.77, df = 1, p = 0.03), the *DAT1 *3'UTR VNTR (χ^2 ^= 12.17, df = 2, p = 0.002) and the *DRD4 *exon 3 VNTR (χ^2 ^= 8.69, df = 3, p = 0.03) (Table 5). In addition, rs998424 in *NET1 *and rs140701 in *5-HTT *displayed an association trend (χ^2 ^= 3.22, df = 1, p = 0.07 and χ^2 ^= 3.24, df = 1, p = 0.07, respectively). rs11568324 was not tested in the AW test due to low minor allele frequency (MAF = 0.01) and subsequent low numbers of informative twin pairs.

Application of a Bonferroni correction to each nominally associated marker for a total of 20 comparisons yielded only the *DAT1 *3'UTR VNTR significant (AW test, p = 0.04).

## Discussion

In this study we investigated the genetic relationship between ADHD symptom scores at two time points in infancy. Consistent with previous reports we found ADHD scores to be highly heritable at age 2 and 3 years, providing evidence for the involvement of additive genetics on the variance of these measures, as well as identifying them as viable measures for molecular studies. Intraclass correlations for our ADHD measure were suggestive of predominantly additive genetic influences at both ages. However, the literature is mixed with regards the effects of dominance and contrast effects, a feature of ADHD that is often found in samples of older children [21]. Dominance and contrast effects are characterized by DZ correlations that are lower than half MZ correlations, and while there is evidence for dominance in symptoms of overactivity in young children [22], there is no evidence for these effects in other studies of activity and attention problems [23]. In light of the power needed to detect dominance and contrast effects [24] and given the lack of evidence for these effects in this study, we did not formally test for them, although future research in large samples using similar measures are needed to clarify this issue.

Phenotypic stability of ADHD symptoms across ages was moderate, producing inter-age correlations of 0.51 - 0.62 (twin 2 - twin 1), which is consistent with previous reports using samples of this age range [10]. The suggestion here is that while symptoms are consistent across ages for the most part, there remains developmental change, which is reflected in the newly emerging additive genetic variance at age 3, a variance component that is unaffected by error associated with fluctuations in evaluations. Prior research has shown a level of genetic stability on ADHD traits across numerous age ranges, including very young children [10,11]. Our analyses concurred with these findings as we found that genetic effects at age 2 are largely shared with those acting at age 3. The suggestion here is that genetic variation that influences variance in ADHD scores at age 2 will be the same as those acting at age 3, on the most part. Having said that, unique effects of additive genetics at age 3 are significant, so while there is substantial genetic continuity across ages, emerging effects cannot be ignored. Unfortunately a limitation of this study was the limited power to assess sex × gene interaction effects in the quantitative analysis. This is an interesting area of research and one that should be considered in future research with more powerful samples, although at present there is little evidence for gene × sex interaction, at least in symptoms of overactivity [22].

Given the results from our quantitative analysis, it is interesting to consider the results of our molecular genetic analyses. At age 2, we found modest, nominally significant (p < 0.05) associations with four variants (*DAT1 *3'UTR VNTR, rs11568324, rs3785157 and rs998424). Although there were some associations in common at age 3 (*DAT1 *3'UTR VNTR and rs3785157), the association between ADHD scores and rs11568324 at age 2 did not replicate at age 3. Further, an age-3-specific association was observed with the *DRD4 *exon 3 VNTR and one SNP in *5-HTT *(rs11080121), findings that are consistent with our quantitative genetic results. Although suggestive at this stage, these findings highlight problems of age-specific genotypic effects that may occur in demographically heterogeneous samples. We may speculate that these differences in genetic association are due to new effects emerging at age 3, implying developmental specificity in which phenotypic consequences of DNA polymorphisms are effectively masked until a particular developmental stage is reached. There are, however, alternative explanations. It might be that subtle differences in ratings between ages causes some manner of spurious association at either age independently, an issue that relates largely to the power of the sample and increases the chance of type I and II errors. In any case, from our analyses it is apparent that there are age-specific effects of genotype on ADHD symptom scores and is thus a factor that should be considered in genetic studies.

An interesting comparison to be drawn is one between this study and an analysis carried out by Mill *et al*. [9], who conducted a similar analysis in a population-based twin sample. Although they used a composite measure of ADHD symptom scores across 2, 3, 4 and 7 years for the main analysis, they also reported some individual time-point data. *DAT1 *was found to be associated with ADHD symptoms at ages 2 and 3, and our report therefore serves as a replication of these findings.

A further point for discussion is the observed difference between the AT and AW tests of association. At age 2, rs3785157 and rs998424 were significantly associated (nominal p < 0.05) only in the AW test. Given the increased power of the AT test to detect association in the absence of stratification, these results may be surprising, and may reflect between-family differences in child ratings. We are, however, unable to assign this observation to any stratification effects because of a non-significant finding in the AP test. This raises issues regarding the power of the sample to detect stratification and makes it difficult to conclude that there are in fact any significant differences in the between and within family components of association. However, of interest is that at age 3, larger discrepancies in effects of these two markers were observed between the AT and AW tests, an observation that is apparent in the AP test which displays significant evidence of stratification. This phenomenon is also seen for associations with the *DAT1 *3'UTR VNTR and *DRD4 *VNTR at age 3, where there is a decrease in p-value in the AW compared to the AT test, albeit with no significant difference in the AP test. Taken together, we conclude that there is evidence for stratification effects, an observation that is not unique to this study [9] and which may reflect between-family differences in rating styles. In particular, it is interesting to note that the pattern of *DAT1 *3'UTR VNTR associations in this study are the same as those observed by Mill *et al*. [9]. Both studies display greater significance for the AT than AW test at age 2, with the reverse effect at age 3. The suggestion is, therefore, that there may be new stratification effects emerging at age 3 that could contribute to the observed age-specific genotypic effects.

A major limitation of this study is the power of the sample to detect genetic association, especially if we consider convincing levels of significance to be in the order of p < 5 × 10^-7 ^[25]. Using the genetic power calculator http://pngu.mgh.harvard.edu/~purcell/gpc/ we estimated that the sample had 47% power to detect a QTL affecting 1% of the phenotypic variance and 71% power to detect a 5% QTL. Despite being underpowered, we detected nominal significance for a number of polymorphisms at ages 2 and 3, and although we cannot rule out the possibility of false positives, the study serves as a proof of principle, in that age-specific effects of genotype on behavioural measures is an issue to be addressed, especially in underpowered samples.

In this study we investigated the genetic relationship between ADHD symptom scores at age 2 and age 3. Although we found that the majority of genetic effects were shared across ages, there was room for some age-specificity. These inferences were borne out in the molecular genetic analyses, whereby associations seen at age 2 replicated at age 3. However, some observed associations were age-specific, which highlights this issue as an important one to consider in genetic association studies.

## Conclusions

This report indicates that although the majority of genetic effects on ADHD symptom scores at age 2 are stable through to age 3, there remains significant emerging effects. As well as enabling us to better understand how genes contribute to the aetiology and origin of ADHD, the report also serves to highlight the importance of demographic homogeneity in molecular genetic studies.

## Conflict of interests

The authors declare that they have no competing interests.

## Authors' contributions

NI carried out the VNTR genotyping, data analysis, and interpretation and drafted the manuscript. KS designed the study, carried out data collection, helped with interpretation and helped draft the manuscript. PA helped with interpretation and helped draft the manuscript. All authors read and approved the final manuscript.

## Pre-publication history

The pre-publication history for this paper can be accessed here:

http://www.biomedcentral.com/1471-244X/10/102/prepub
